# Dr. Ralph Walsh’s Call Book

**Published:** 1876-05

**Authors:** 


Dr. Ralph Walsh's Combined Call Book and Tablet.—Of all the call books and physician's pocket records that we have seen, this one more perfectly fills the "*indication*" than all others. In the first place, its size is more convenient—it is longer and much thinner than those in use. Then the dates are so arranged that it may be used until full, commencing at any time during the year. The tablet is certainly quite an addition. In addition to all the information in regard to antidotes, the rare or poisonous remedies, he gives a complete list with doses of all the important remedies. This valuable little book can be had by application to Dr. Ralph Walsh, Washington, D. C. Price, $1.50.

